# Outcomes of external cephalic version for antenatal women with breech presentation in a secondary hospital in Vellore, Tamil Nadu - a retrospective review

**DOI:** 10.4274/jtgga.galenos.2020.2020.0140

**Published:** 2020-12-04

**Authors:** Tobey Ann Marcus, Shalini Jeyapaul, Sam Marconi David, Dimple Jamkhandi, Anne George Cherian

**Affiliations:** 1 Department of Obstetrics and Gynaecology, Christian Medical College and Hospital, Vellore, India; 2 Department of Community Medicine, Christian Medical College and Hospital, Vellore, India; 3 Department of Family Medicine, Christian Medical College and Hospital, Vellore, India

**Keywords:** Breech presentation, external cephalic version, limited resource setting

## Abstract

**Objective:**

Breech presentation is the most common fetal malpresentation at term, with an incidence of 3-4%. External cephalic version (ECV) is a procedure that can be offered to women with breech presentation beyond 36 weeks of gestation to convert it to cephalic presentation, reducing the risks of a vaginal breech delivery and the morbidities associated with caesarean section.

**Material and Methods:**

We retrospectively reviewed the records of women who underwent ECV between October 2012 and June 2020 with the objectives of determining the success rate of the procedure, the mode of delivery, the maternal and neonatal outcomes, periprocedural complications and their management.

**Results:**

Among the 200 women who underwent the procedure with a 64% success rate (128 women), there were 110 vaginal deliveries (56.7%) including five vaginal breech deliveries, and 84 women (43.2%) underwent caesarean section, which included 24 women who had successful ECV but needed emergency caesarean for other indications. There was no significant difference in the neonatal APGAR scores in those who had a successful ECV and those who did not. Only three women (1.5%) experienced any significant periprocedural complication.

**Conclusion:**

These results suggest that ECV improves the possibility of a vaginal delivery with an overall low complication rate, reducing the neonatal risks associated with vaginal breech delivery and the maternal morbidity of a caesarean section. It may thus contribute to reducing the primary caesarean section rate, making it a useful intervention, especially in limited resource settings.

## Introduction

Breech presentation is the most common fetal malpresentation with an incidence of about 3-4% at and near term (1). It could be secondary to a pre-existing maternal or fetal abnormality, or related to abnormal placentation, such as placenta praevia, cornual location of the placenta, or could also be a chance occurrence. Whatever the cause for the malpresentation, breech presentation is associated with an increased risk of either a complicated vaginal delivery with significant risk of perinatal morbidity or mortality, or a caesarean section which is accompanied by increased risk of maternal and fetal complications.

The term breech trial which was undertaken by Hannah et al. (2), reported a significantly decreased perinatal mortality and morbidity following planned caesarean section when compared to planned vaginal breech delivery. Following the publication of these results, a shift occurred in the management of breech presentation in labour, towards caesarean section with very few individuals and institutions being willing to take risks with planned vaginal breech delivery. This resulted in an increase in the caesarean section rate for breech presentation to 95% internationally (3).

In a Cochrane review, published in 2003, comparing planned elective caesarean section with a trial of vaginal delivery for women with breech pregnancy at term, it was found that an elective caesarean section had increased maternal morbidity when compared to vaginal delivery. Furthermore, 45% of the women planned to have a trial of vaginal delivery eventually had an emergency caesarean section for another indication (4). Other studies have also shown that, although there is a minimal risk overall, the incidence of maternal mortality associated with planned caesarean section is higher than that of vaginal birth (5,6). Apart from the increased risk of morbidity and mortality in the index pregnancy, caesarean delivery also poses significant risks for subsequent pregnancies, including the possibility of placenta praevia and morbidly adherent placenta. Another factor against advocating universal elective caesarean delivery for breech presentation is that the procedure requires the expertise of an obstetrician or another surgically trained health worker. This limits the role of low-risk obstetric health workers like midwives and general practitioners.

A review of studies looking at strategies to reduce global caesarean section rates demonstrated that external cephalic version (ECV) was the only significant clinical intervention to reduce primary caesarean sections (7). However, despite these evidence-based benefits, it was found to be an underused procedure, resulting in a loss of skill over time in performing this procedure.

ECV is a procedure by which the singleton fetus is gently manipulated externally from a non-cephalic to a cephalic presentation. This is carried out after 36 weeks of gestation and usually after the administration of a tocolytic agent to relax the uterus. The purpose of this intervention is to decrease the incidence of breech presentation in labour and to decrease the maternal morbidity and mortality associated with caesarean delivery. The success rate of ECV is approximately 65% at term to convert non-cephalic into vertex presentation (8).

International societies of obstetrics and gynaecology have issued guidelines recommending the use of ECV in term antenatal women with the fetus in breech presentation. Absolute contra-indications to the procedure include multiple gestations, rupture of membranes, antepartum haemorrhage in the antecedent week, abnormal cardiotocography, the presence of uterine anomalies, fetal hyper-extended head and any other condition which otherwise warrants caesarean delivery, such as placenta praevia (9). In the absence of contra-indications, ECV should be offered to all women with a non-cephalic presentation beyond 36 weeks of gestation and should always be performed by skilled personnel (obstetrician or trained midwife).

The diagnosis of breech presentation on clinical examination during antenatal reviews is important because, in that event, the option of ECV can be considered and discussed with the patient. If a breech presentation is undiagnosed during the antenatal check-up, the woman usually presents in labour or with pre-labour rupture of membranes and in most cases is taken for emergency caesarean section, unless she is close to spontaneous vaginal breech delivery at the time of admission to the labour room.

This study was undertaken to investigate the feasability of ECV as an option in women with non-cephalic presentation at term, especially in a secondary hospital and other resource-limited settings, to avoid the neonatal morbidity associated with vaginal breech deliveries and the maternal and neonatal morbidity associated with caesarean delivery.

The objectives of this study were, firstly, to determine the success rate of ECV and the distribution of mode of delivery among women who underwent the procedure. Secondly, to study the delivery outcomes (maternal and neonatal) of women who had successful ECV and finally, to enumerate peri-procedural complications encountered and their management.

## Material and Methods

This is a retrospective review of women who were diagnosed with breech presentation and underwent ECV at our hospital from October 2012 to May 2020. Clearance was obtained from The Institutional Review Board and Ethics Committee CMC Vellore, (IRB number: 11412). Informed consent couldn’t be obtained in view of the retrospective nature of the study.

### Setting

The study was based in a 140-bedded, secondary care level hospital, under the department of Community Health of a multidisciplinary tertiary care centre in South India. The hospital has been providing primary and secondary level maternal and child health services, focussing primarily on the residents of Kaniyambadi block, which is a rural development block in Vellore district, for the past 40 years. The services also extend to the surrounding areas of Vellore town as well as to residents of adjoining districts who wish to seek care at the hospital. Another area of primary focus is the tribal population of the Jawadhi hills which span over four panchayats in Vellore district and 11 in Thiruvanamalai district. Both out-patient and in-patient facilities are available at the hospital for different health conditions. The out-patient clinic includes both general services and speciality clinics for maternal and child health as well as communicable and non-communicable chronic diseases.

Obstetric services can be availed by anyone registered in the antenatal clinic at the base hospital or in the various mobile clinics that cater to the residents of Kaniyambadi block and the Jawadhi hills. The maternal health related services provided include a 24-hour labour room facility for normal and assisted delivery, operation theatre and caesarean sections under spinal anaesthesia. There is an established referral system to the department of obstetrics and gynaecology at the affiliated tertiary level centre. Data concerning deliveries, associated risk factors, and birth outcomes are recorded during pregnancy and childbirth in out-patient as well as in-patient records and entered into an electronic database. This electronic database with respect to both base hospital and community data has been maintained over the last 25 years.

In our hospital, ECV is offered to women with non-cephalic presentation at term in whom there are no contra-indications for vaginal delivery or for the procedure itself.

### Time period

Over the last eight years (from 2012 onwards), a register has been maintained of women with breech presentation who underwent ECV. This record was used to retrieve information from the electronic database, of the outcomes of women who underwent the procedure from October 2012 to May 2020, including mode of delivery and condition of the baby at delivery.

### Procedure

During the antenatal out-patient review, if a non-cephalic presentation was diagnosed at a gestational age of 36 completed weeks or more, the woman was counselled regarding ECV if no contra-indications for ECV were present. Women who consented to the procedure were admitted to the ward, and an ultrasound was done to determine the estimated fetal weight, amniotic fluid index, type of breech (flexed/extended), the position of the head (whether hyperextended or not), the position of the placenta and to rule out uterine anomalies. If there were no further contra-indications identified by ultrasound, the procedure was carried out after administration of a tocolytic agent (inj. terbutaline 0.25 mg, subcutaneously). ECV was carried out by the obstetrician, either by the backward flip or forward roll technique. The fetal heart rate was checked before, during and after the procedure using ultrasonography. Following the procedure, bradycardia or non-reassuring fetal heart rate patterns were ruled out, and the woman was observed for rupture of membranes, the onset of labour pains or decreased fetal movements, following which she was discharged from the hospital. After a successful ECV, the patient was followed up regularly in the antenatal clinic, and if she did not go into spontaneous labour, was induced past dates, as per standard of care in our setting. Emergency caesarean delivery was performed for obstetric indications. If, however, the procedure was unsuccessful, in the absence of any contra-indications, the option of vaginal breech delivery was discussed, providing knowledge about benefits and possible complications, to help the patient make an informed decision. If vaginal breech delivery or ECV was contra-indicated, or the woman was unwilling for these procedures, she was planned for an elective caesarean delivery.

### Inclusion criteria

All women who presented with a non-cephalic presentation at term and underwent an attempt at ECV were included in the study, and their information was retrieved for analysis.

### Statistical analysis

Data was transferred to Microsoft Excel, and statistical analysis was done using SPSS, version 24.0 (IBM Inc., Armonk, NY, USA). Continuous variables were tested for normality using Kolmogorov-Smirnov (K-S) test and Shapiro-Wilk test and continuous variables that were not normally distributed were expressed as median and range and discrete variables as frequencies and proportions. The associations were determined using chi-square test, and a p-value of <0.05 was considered to be statistically significant.

## Results

**Figure 1:**
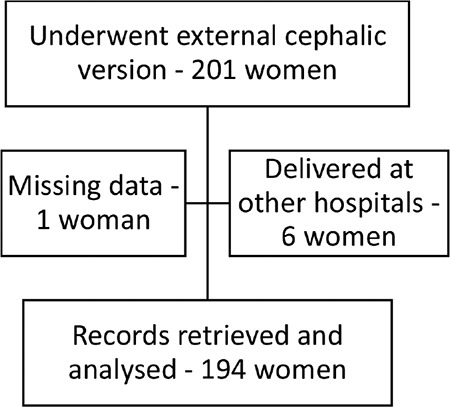
Participants flow diagram

As depicted in Figure 1, a total of 201 women were documented in the register as having undergone an attempt at ECV between October 2012 and June 2020. Of these, one woman had entirely missing data, and six women delivered at other hospitals, hence their delivery records were not available. For the remaining 194 women, baseline demographic information and delivery details were extracted from the hospital electronic database and analysed (Table 1).

Of the women included in the study, 176 of them were within the age group of 20 to 35 years (90.7%), median (range) age being 24.08 (20) years. Vaginal deliveries comprised 56.7% (110 women) of the total number of deliveries, of which five were vaginal breech deliveries. There were 84 women (43.2%) who underwent caesarean section for various indications including the elective cases who had an unsuccessful ECV. Among the babies delivered in the study group, 77.3% (150 babies) had normal birthweights of 2,500 to 3,500 gm, 9.3% (18 babies) were large for gestational age and 12.9% (25 babies) were low birth weight. One baby (0.5%) was in the very low birth weight category (<1,500 gm). There were 81 male babies and 113 female babies (41.8% and 58.2%, respectively) and 98.9% of the babies were healthy, i.e. normal APGAR score, at the time of delivery (192 out of 194 neonates).

The procedure was observed to be successful in 64% of patients (128 out of 200 women). The denominator for the success rate of ECV calculation was taken as 200 women since the data for immediate outcome of the procedure (i.e. successful/unsuccessful ECV) was available for them. The 6 women who delivered at other hospitals were excluded from the analysis for associations or delivery outcomes. From the available data, associations were studied between maternal age, parity, gestational age at delivery and birth weight of the baby to study factors that could affect the success of ECV. The findings are presented in Table 2.

**Figure 2:**
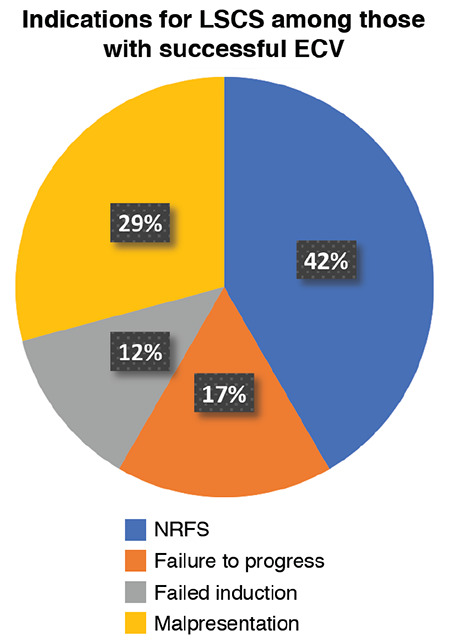
Indications for caesarean section among women who had successful external cephalic version (n=24) ECV: External cephalic version, LSCS: Lower segment caesarean section, NRFS: Non-reassuring fetal status

Among the analysed data of women who had successful ECV (n=123), 99 of them (80.4%) delivered vaginally and 24 underwent caesarean section (19.5%). The indications for caesarean section (Figure 2) were: non-reassuring fetal status (n=10), failure to progress (n=4) or failed induction of labour (n=3) and malpresentation (n=3 transverse lie; n=3 breech in labour; and n=1 persistent mentoposterior).

In those for whom ECV was unsuccessful, 11 women (15.4%) delivered vaginally including four vaginal breech deliveries, three suction cup deliveries and four normal vaginal deliveries following spontaneous version. The remaining 60 women (84.5%) were delivered by caesarean section.

For the neonatal outcomes, over 95% of the babies had a normal APGAR at the time of delivery, regardless of success or failure of the procedure, including 122 babies (99.2%) among the successful ECV group and 70 babies (98.5%) among the unsuccessful ECV group. One baby had a 5-minute APGAR score <7, born to a mother in the unsuccessful ECV group, who presented later with a cephalic presentation in labour and had non-reassuring fetal status in second stage, delivered normally with episiotomy. The baby was referred to the tertiary care hospital for therapeutic cooling in view of hypoxic ischemic encephalopathy and is now doing well with normal developmental milestones and being followed up in the outpatient department. There was one intrapartum stillbirth in the successful ECV group, in a woman who was diagnosed to have breech presentation at admission for induction of labour. She underwent ECV with no peri-procedural complications, followed by pre-induction cervical ripening with prostaglandin E1 as per standard of care. She complained of decreased fetal movements the next day and was found on ultrasound to have intra-uterine fetal demise. The baby was born through meconium stained amniotic fluid and there was no other evident cause for the stillbirth identified following delivery including no evident growth restriction or external anomalies.

Only three women had significant procedure related complications. One had persistent severe variable decelerations on non-stress test, warranting an immediate caesarean section, one had pre-labour rupture of membranes, and one woman had a placental abruption. This latter patient had a successful ECV, reactive post-procedure non-stress test and was discharged from hospital to review in the OPD. In addition, she was instructed to report to the labour room in case of any complications, which were explained to her in detail. She presented to the labour ward eight hours later with antepartum haemorrhage, and a diagnosis of placental abruption with non-reassuring fetal status was made, for which she underwent an emergency caesarean section. In all three cases, there were no adverse neonatal outcomes, and the babies all had normal APGAR at delivery and an uneventful neonatal period.

## Discussion

This retrospective review was undertaken with the aim of determining the success rate of ECV for women presenting with a non-cephalic presentation at term. A further aim was to study both maternal and fetal delivery outcomes of these women, and to see if there were any significant peri-procedural complications.

The success rate of ECV was found to be 64%, which is comparable to the success rate of approximately 60-65% from other reported international and national data (8,10). From a previous meta-analysis done in 2008, it was established that certain factors, such as multiparity, gestational age, non-engagement of the presenting part and administration of a tocolytic were specific clinical factors that predicted successful ECV (11). In our study group, all patients were administered a tocolytic and there was no documentation of engagement/non-engagement of the presenting part in our records. Multiparous women were found to be more likely to have a successful procedure (75% multiparous vs 55.3% primiparous) and this difference was statistically significant. Although the recommendation is to perform ECV by 36 completed weeks in primigravidae and 37 completed weeks in multigravidae, the gestational age at the time of ECV was not found to be significantly associated with success or failure of the procedure (Table 2).

A successful ECV also had a positive impact on the mode of delivery with a majority of these women delivering vaginally, with a cephalic presentation (80.5%) compared to those who had an unsuccessful ECV, with only 15.5% vaginal deliveries. This outcome was statistically significant with p<0.001 and an odds ratio of 22.5. It should also be noted that among those who had an unsuccessful ECV, seven out of 11 women eventually presented as cephalic and delivered vaginally. This emphasises the possibility of spontaneous version, even after a failed ECV attempt, and the importance of rechecking and making a correct diagnosis of presentation, even if the woman is admitted for a planned caesarean following “failed ECV”.

The women who had a successful ECV were significantly more likely to deliver vaginally compared with those who had an unsuccessful ECV (p<0.001). Only 19.5% of women who had a successful ECV needed a caesarean section for delivery (Table 3). This is comparable to the findings of another study from Hong Kong, which found 19.7% women who underwent caesarean deliveries following successful ECV (12). The caesarean deliveries in the ECV success group with a vertex presentation was only 13.8% compared to the 24% rate reported by Stine et al. (13).

Among the neonatal outcomes, there was a slightly higher proportion of perinatal morbidity (5-minute APGAR score <7)/mortality, following unsuccessful ECV compared with successful ECV (1.4% vs 0.8%, respectively) but this difference was not significant (Table 4). A similar study done in a rural tertiary care hospital in Maharashtra reported all babies to have APGAR of 9 at 5 minutes and no fetal complications or deaths attributable to ECV (14). No difference in perinatal outcome has been seen in other higher-powered studies either, rather showing comparable outcomes in both groups (15). The overall low peri-procedural complication rate observed (1.5%) is consistent with findings across other studies (15,16,17). We observed one minor (prelabour rupture of membranes) and two major (one placental abruption and another fetal distress requiring immediate caesarean delivery) procedure related complications, as discussed earlier.

### Study limitation

The chief limitation of this study is the retrospective design, due to which much data, as well as additional parameters that could have affected the outcome, were not retrieved. We were not able to study the association between success of the procedure and other factors such as estimated fetal weight and other ultrasound features including type of breech, amniotic fluid index at the time of ECV and maternal body mass index, due to incomplete data. At the beginning of the study period, the out-patient charts and discharge summaries for all in-patients were manually hand-written records which were archived in the medical records department, making them difficult to retrieve for additional relevant information. During the latter part of the study, the medical information entry format became digital with scanned out-patient charts and online discharge summaries. Due to this change in the data entry format, there was a lack of uniformity in data availability.

## Conclusion

The results of this study suggest that ECV is a useful procedure in women with breech presentation at and near term, with no contra-indications for a vaginal delivery. Converting the fetal presentation to cephalic gives the opportunity for a normal vaginal delivery, thereby reducing the neonatal morbidity of a vaginal breech delivery and the maternal morbidity of a caesarean section. If the option of ECV for breech presentation was not considered, the majority of those women would have undergone a primary caesarean section. Therefore, this procedure has a definite role in reducing the number of primary caesarean sections and also has a low overall complication rate, making it a useful tool for obstetric management, especially in limited resource settings.

## Figures and Tables

**Table 1:**
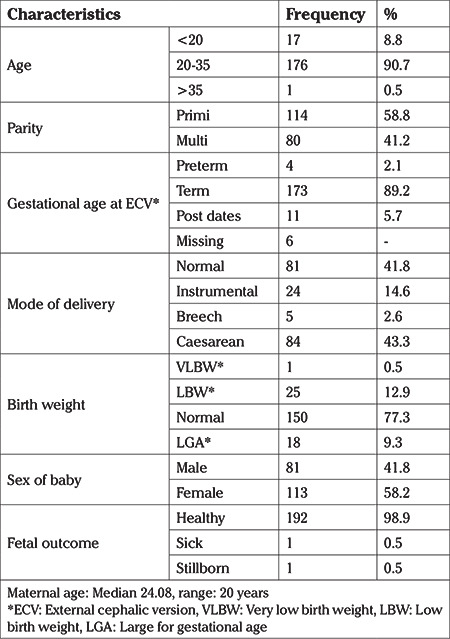
Baseline characteristics

**Table 2:**
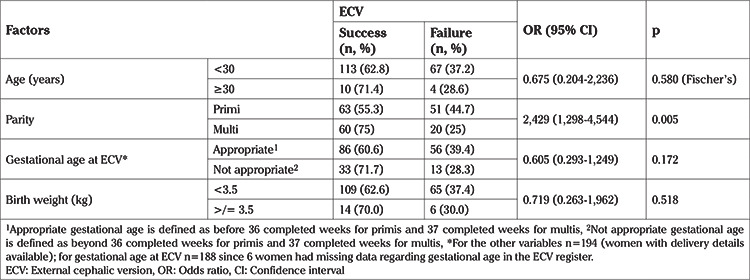
Factors affecting the success of external cephalic version

**Table 3:**
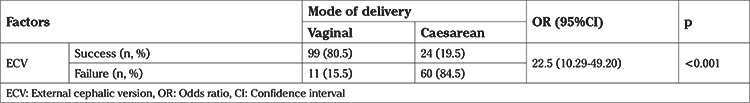
Association between external cephalic version success and mode of delivery

**Table 4:**
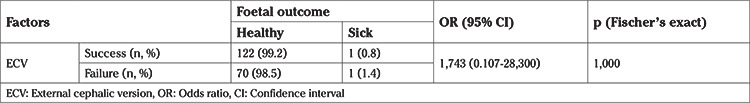
Association between external cephalic version success and neonatal outcome
